# The impact of students and curriculum on self-study during clinical training in medical school: a multilevel approach

**DOI:** 10.1186/s12909-016-0846-3

**Published:** 2017-01-13

**Authors:** J. Barbosa, A. Silva, M. A. Ferreira, M. Severo

**Affiliations:** 1 Department of Simulation and Medical Education, Faculty of Medicine of the University of Porto, Al. Prof. Hernâni Monteiro, 4200-319 Porto, Portugal; 2 Undergraduate Education Department, Institute of Biomedical Sciences Abel Salazar of the University of Porto, Porto, Portugal; 3 Department of Simulation and Medical Education, Faculty of Medicine of the University of Porto, Porto, Portugal

**Keywords:** Clinical training, Curriculum design, Medical students, Self-directed learning, Self-study

## Abstract

**Background:**

In higher education, the focus has shifted from the acquisition of knowledge to learning objectives and skills. This means that, the majority of student learning time is spent independently working outside the classroom. Students take an active role in setting goals, deciding how to achieve them, and planning individual study time. Although extensive research has recognized the importance of curriculum and students’ characteristics in time devoted to self-study, it is still unclear to what extent these variables affect time to study. Due to the growing reliance on self-directed learning in medical education, and in an attempt to elucidate this issue, this research aims to evaluate self-study time during clinical training and assess whether this is more influenced by the student or the curriculum.

**Methods:**

A questionnaire was given to 1220 medical students (43.3% of the enrolled students). The students were asked to indicate the average number of study hours per week beyond the time allocated to classes for each clerkship (rotation) attended. Variation and generalizability of students’ self-study were estimated using linear mixed models.

**Results:**

Findings showed that the intrinsic differences within students were a greater source of variation in self-study time than differences within clerkships (56.0% vs. 6.9%). If the amount of self-study dedicated to an individual clerkship is to be determined, at least 32 students are needed to achieve acceptable reliability. However, this data with two clerkships per student can used to retrospectively measure the self-study reported by students in clinical training.

**Conclusions:**

The findings suggest that, both, curriculum and student characteristics influence self-study in undergraduate clinical training. Indeed, students’ characteristics play a significant role in time devoted to study. Further research should be undertaken to investigate students’ characteristics that may predict self-study during undergraduate medical training.

## Background

Workload is measured as the number of contact hours (timetabled study hours, i.e. class attendance) plus the time spent on self-study (non-timetabled study hours, i.e. time spent outside the classroom for learning purposes, either for class assignments, group work, or studying for an assessment) [1]. As contact hours can be easily identified and account for approximately one-third of the total working time [2], it remains important to find out the time students devote to self-study. In higher education, the focus has shifted from the acquisition of knowledge to learning objectives and skills. The majority of student learning time is spent independently working outside the classroom. This educational approach emphasizes the active role of students in setting goals, deciding how they will achieve them, and scheduling and planning one’s study time [3]. Consequently, to a large extent, it depends on students’ time allocations for self-study.

For many students the amount of work required is the most important factor affecting their involvement with a course [4]. To learn effectively, it is essential that students are given sufficient time for processing their learning [5]. However, there is typically no attempt to enforce the hours of self-study, and it is simply assumed that these will take place in the program. Within this framework, monitoring student’s self-study and emphasizing its importance is key to the learning process.

Factors found to be influencing time to study have been explored in several studies. Published research recognizes that curriculum characteristics affect the amount of time students devote to study. For example, factors such as difficulty of the scientific area, field of study, number of assessments and different supervised learning environments are considered to be associated with time devoted to self-study [6–11]. Conversely, students differ in the time they need to achieve learning outcomes, which can be explained by individual characteristics and learning styles [6, 12–16]. For medical students, self-learning experiences and time for self-study will allow them to develop skills for lifelong learning. In clinical training where the main concern is patient care, students are faced with less structured styles of teaching and learning [17, 18]. The tasks to be learned require simultaneous integration of multiple sets of knowledge, skills and behaviors at a specific time and place [19]. The strategic selection and application of learning skills is crucial to the adaptation of students to this challenging learning environment. Some students find the environment motivating and exciting, whereas others become stressed and perceive a high workload.

Although extensive research has recognized the importance of curriculum and individual differences in time devoted to self-study, it is still unclear to what extent these variables affect time to study [20]. Given the responsibility of schools to develop adequate curricula for achieving learning objectives, it is important to discriminate the relative contributions from students and curriculum to the time devoted to self-study. To our knowledge, no study has explored this issue within clinical training. The present research explores, for the first time, the variation in time devoted to self-study during clinical training as accounted for by student and curriculum factors. We also aimed to use variance estimates to determine the optimal number of clerkships needed to estimate students’ self-study and, in turn, the optimal number of students needed to estimate each clerkship’s self-study. These figures are needed because resources are limited, and in medical education it is not always easy to get volunteer students to answer questionnaires [21]. The results may inform decisions about how best to allocate self-study across clerkships and guide teachers about where to direct their efforts to minimize individual differences and enhance the effectiveness of self-study.

## Methods

### Educational context (curriculum outline)

This study was conducted in a public medical school in North of Portugal, with a curriculum that complies with the Bologna Process of 2007/2008. One ECTS (European Credit Transfer and Accumulation System) is defined as a total of 27 h, and a full year of academic study would yield 60 ECTS, representing a workload of 1620 h. Timetabled study-related activities constitute 25–35% of the total number of working hours. The undergraduate medical course lasts for 6 years consisting of basic/pre-clinical work (first 3 years) and a clinical stage following that. The first 3 years cover 32 semester disciplines of basic and pre-clinical sciences. The last 3 years cover 25 clerkships of clinical practice attended one at a time throughout the academic year. In addition, students develop a research project and take an optional course (listed in Appendix).

### Study design

As part of a program evaluation at the end of the assessment period, students in clinical years completed a questionnaire to evaluate each clerkship. The questionnaire comprised one item about time devoted to self-study per week: How many hours did you spend on average per week on self-study (study outside the hospital for learning purposes whether it is for class assignments, group work, or studying for an assessment) throughout the academic year?

The data comprised input for the 25 clerkships assessed in four consecutive academic years (cohorts 2008/2009 till 2011/2012). A total of 1220 students of the 2820 enrolled (43.3%) participated in the study (408, 276 and 536 from 4th, 5th and 6th years, respectively). Each student answered based on all their clerkships of the respective curricular year: nine clerkships in year 4, ten in year 5, and six in year 6 (Median per curricular and academic year = 69; Percentile 25–75 = 52–141).

### Definition of exposures/outcomes

Our outcome was the self-study hours per week devoted to each clerkship. Self-study time was expressed in hours/week. Our main exposures were clerkships and students. We also considered if self-study hours allocated in our curriculum and academic year explained variability of our main exposures. The self-study hours allocated in our curriculum were defined by ECTS*27*SH and were scaled by subtracting the mean of allocated study hours of sample: 24.9, where SH is the percentage of study hours allocated in our curriculum to self-study hours.

### Statistical analysis

Differences between self-study hours devoted to each clerkship were described with mean and standard-deviation (SD).

Linear mixed effects models were used to estimate the relative contribution that clerkships and students made to the variation in self-study time. The variables, self-study hours allocated in our curriculum and academic year, were entered as fixed effects of the linear mixed effects models. To account for intra-students and intra-clerkships variance in the data we performed the multilevel analysis using student-level and clerkship-level, as random effects of our model.

Three models were constructed. The first model, an empty or zero model, was built only with the intercept as a fixed effect and random effects for clerkships and students. The second model contained the self-study hours allocated in our curriculum/week as the fixed effect. The third model additionally included the academic year. To compare the fit between models, we used the log likelihood ratio test, the Bayesian information criteria, and Akaike information criterion.

The fixed effects were expressed as regression coefficients and corresponding 95% confidence intervals (CI). The random effects (measures of variation) were expressed as Intraclass Correlation Coefficient (ICC) and proportional change in variance (PCV). The ICC provides an estimate of the proportion of total variance related to students or clerkships. The PCV assessed the amount of variance explained. The PCV was calculated as:

$PCV=\frac{V_0\mathit{\hbox{-}}{V}_i}{V_0}\times 100$,where V_0_ is the estimate of the initial (null) variance of empty model and V_i_ is the variance of model adjusted for curriculum variables added to empty model [22].

The data from the variance components where then used in a decision study (D study) to estimate the optimal clerkships needed to estimate students’ self-study and the optimal students needed to estimate each clerkship’s self-study. For this, we required a reliability of 0.7. Statistical analyses were performed using R software version 2.14.1. For all comparisons, statistical significance was set at α level ≤0.05.

## Results

### Curriculum and student effect on self-study hours

We observed differences between the average self-study hours devoted to each clerkship. The least amount of time spent was 5.1 h per week (SD = 5.0) at Bioethics and Deontology in fourth curricular year. The greatest amount of time spent on self-study was 19.2 (SD = 12.4) hours per week in the Medicine rotation of 5th year (Table 1).

**Table 1:** Number of hours spent per week on self-study for each clerkship

Year	Clerkship	Mean (standard-deviation)
4th	Bioethics and Deontology	5.1 (5.0)
	Dermatology and Venereology	13.0 (11.4)
	Medicine I	14.8 (12.6)
	Neurology and Neurosurgery	9.9 (8.0)
	Orthopaedics and Traumatology	9.1 (7.1)
	Otorhinolaryngology	7.5 (6.6)
	Radiology	12.2 (10)
	Surgery I	13.1 (11.2)
	Therapeutic	11.3 (9.3)
5th	Anesthesia	8.1 (7.1)
	Legal Medicine	8.3 (6.7)
	Medicine II	19.2 (12.4)
	Opthalmology	10.2 (8.1)
	Obstetrics and Gynecology	12.6 (10.7)
	Pathological Anatomy	16.4 (10.5)
	Pediatrics I	12.7 (10.2)
	Psychiatry I	10.2 (7.7)
	Surgery II	12.0 (10.2)
	Urology	12.0 (9.4)
6th	Community Medicine	10.1 (10.8)
	Medicine II	13.6 (11.9)
	Obstetrics	8.3 (9.5)
	Pediatrics II	8.0 (9.2)
	Psychiatry II	9.2 (10)
	Surgery III	9.4 (8.9)

**Fig. 1:**
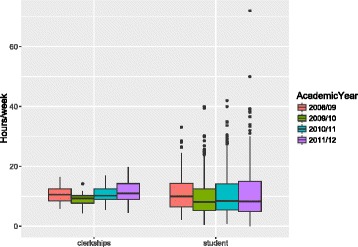
Average time spent in hours of self-study per week

Figure 1 shows the average self-study per week by academic year and the average clerkship’s self-study per week by academic year. There is greater individual variation in time spent on self-study by students than by clerkship.

Table 2 presents the results of the mixed linear models, with time devoted to self-study as the outcome. Students spent an average of 9.8 (95% Confidence Interval: 9.0–11.2) hours per week in each clerkship in clinical training. For each hour of increase in the allocated self-study hours, the reported self-study hours increased 0.2 (95% Confidence Interval: 0.1–0.3), i.e., for every hour of allocated self-study, students dedicated an additional 12 min to self-study. A non-significant effect for the academic year (Model 3) was observed.

**Table 2:** Fixed and random effects in self-study hours

	Model 1	Model 2	Model 3
Source of Variation	Coefficient (95% CI)	Coefficient (95% CI)	Coefficient (95% CI)
Fixed effects			
(Intercept)	9.8 (9.0;11.2)	9.8 (8.9;11.0)	10.6 (9.1;12.2)
Allocated Self-Study Hours	–	0.2 (0.1;0.3)	0.2 (0.1;0.3)
Academic year	–	–	
2007/2008			Reference
2008/2009			−1.5 (−2.7; −0.3)
2009/2010			−0.8 (−1.7;0.7)
2010/2011			−0.4 (−1.1;1.2)
Random effects			
Clerkship			
Variance	7.3	7.3	7.3
PCV (%)	Reference	0.0	0.0
ICC (%)	6.9	6.9	6.9
Student			
Variance	59.3	59.2	59.1
PCV (%)	Reference	0.2	0.3
ICC (%)	56.1	56.0	55.9
Residual variance (Clerkship*Student)	39.2	39.2	39.2
*p* value ^+^	–	<0.001	0.268
AIC	57,663	57,654	57,656
BIC	57,691	57,689	57,712
Log L	−28,827	−28,822	−28,820

*Abbreviations*: *PCV* proportional change in variance, *CI* confidence interval, *ICC* intraclass correlation coefficient, *AIC* Akaike information criterion, *BIC* Bayesian information criteria, *Log L* Log likelihood

^+^ Likelihood ratio test

**Fig. 2:**
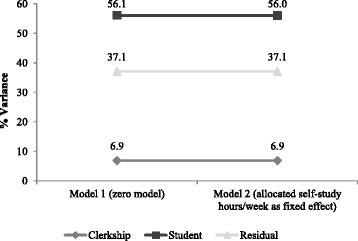
Variability explained by clerkships and students in the self-study hours

In Model 1 only 6.9% of variance of the total observed self-study hours can be ascribed to differences within clerkships. In contrast differences within students accounted for 56.1%. After adjusting for allocated self-study hours (Model 2) the variance within clerkships and within students remained the same. The residual variance between clerkships and students accounted for 39.2 (37.1%) of the variance of total observed self-study hours (Fig. 2).

### Reliability

**Fig. 3:**
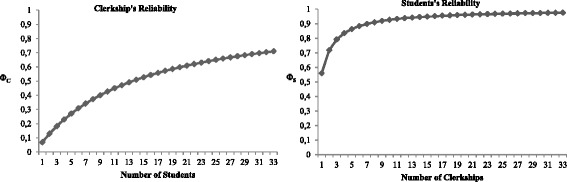
The clerkship’s reliability by the number of students and student’s reliability by the number of clerkships

Figure 3 shows the progression of the reliability coefficients in terms of the number of clerkships needed to determine students’ self-study, and the number of students needed to discern self-study in each clerkship in clinical training. If we accept a level of 0.7 on the grounds of feasibility, then 32 students would be needed to achieve reliability for the clerkship. Based on the available estimated variance components, a reliability of at least 0.70 requires two clerkships to achieve reliability for the student.

## Discussion

Due to the growing reliance on self-directed learning in medical education, this study aimed to explore the extent to which students and curriculum influence the time devoted to self-study within an undergraduate clinical training program. The first important finding was that, as expected, time devoted to self-study in clinical training is related to the curriculum and student characteristics. Estimated variance attributable to differences among students (55.9%) is about eight times as large as the 6.9% estimated variance attributable to differences among clerkships. We may be underestimating the variance explained by clerkships because only differences between clerkships were evaluated and not differences between learning environments or supervisors. However, differences between learning environments and supervisors would never exceed the variance explained by students because clerkship student interaction variance was 39.2 (37.1%). This suggests that students’ characteristics are a greater source of variability in self-study than clerkships in undergraduate clinical training. Clearly, this phenomenon is affected by the number of students needed to obtain a reliable estimate of self-study for each clerkship. If we want to know the amount of self-study dedicated to an individual clerkship, we need at least 32 students to achieve acceptable reliability. However, data with two clerkships per student is useful for measuring retrospectively reported self-study in clinical training (D study interpretation).

These findings support Van der Huk et al. who showed large individual variation in the time spent on self-study for each module in a problem-based curriculum [6]. It is encouraging to compare our figures with those of Snelling et al. because together they suggest that effective self-study is not just a matter of time available, but depends on students taking responsibility for their own learning [2]. Beyond timetabled training sessions, students undertake self-study with textbooks, examination guidelines, scientific articles, the Internet, videos, previous OSCEs and practice skill training [20]. Based on our results it would be important to look for students’ characteristics that may predict study time investment in the clinical years. For instance, Wilkinson et al. found that students who have a desire to achieve and who are well organized and committed to their career choice as a doctor will devote more time to study [14]. Students who lacked confidence and had inefficient study habits were also noted to study more [14]. In contrast, students who procrastinated used only a proportion of the self-study time available [11]. Teachers should focus their efforts on supporting students to acquire self-learning skills and guide them to efficiently use self-study time [23].

Our results showed that, in general, medical students appear to be spending 9.8 h/week in self-study in clinical training. This constitutes half of the self-study allocated in the curriculum. Previous studies have assessed the study time spent on activities of clinical training based on student report at the end of an assessment period. Dolmans et al. evaluated the time students spend in different study activities over the course of eight clinical rotations [24]. They found that students devoted 8 h per week to self-study, slightly less than our results. Similar to our findings, Medicine and Pediatric rotations involved more self-study time. In an anesthesiology residence program, the average time spent in self-study was 8 h/week [25], further supporting our results. However, these findings differ from some published studies that record student’s study time over relative short periods with weekday hours spent on each activity. A project designed to describe and analyze a typical student week in clinical practice showed that students studied for about 60 h per week with self-study comprising 26 of these hours [26]. Another study of final year medical students also evaluated a typical week in three clerkships. Median total time spent on learning activities was 43.7 h, and of this about 6.2 were allocated to self-study.

The analysis of ECTS undertaken here, has extended our knowledge of how students distribute their effort in clinical training. There were specific courses for which self-study was generally more demanding. Results showed that as allocated self-study hours increase, the time spent on self-study also increases. For each hour of allocated study, students engaged in a further 12 min of self-study. As shown originally by Jansen et al. [10], time management is easier to control in the clinical years when students have one clinical module at a time.

Finally, a number of important limitations need to be considered. Reliability may be compromised when results are based on subjective information. However, the reduced variation in time devoted to study when using the mixed model with the academic year (cohorts 2008/2009–2011/2012) demonstrates a pattern over time and supports reliability of the results. Furthermore, preliminary studies shown that this method provides a reliable indicator of the time spent on individual study [27, 28]. A second and obvious limitation is that the present findings may not be generalizable to non-medical schools.

## Conclusion

In summary, the findings of this study suggest that, both, curriculum and students characteristics appear to be related to time devoted to self-study. It is proposed therefore that further research should be undertaken to investigate the students’ characteristics that may predict self-study during undergraduate medical training.

## Appendix

**Table 3:** Clinical Practice per curricular year

Curricular Year	Credits (ECTS)
Year IV	
Bioethics and Deontology	3
Dermatology and Venereology	3.5
Medicine I	16
Neurology and Neurosurgery	5
Orthopaedics and Traumatology	5
Otorhinolaryngology	3.5
Radiology	4
Surgery I	15
Therapeutic	5
Year V	
Anesthesia	3.5
Legal Medicine	3.5
Medicine II	13.5
Ophthalmology	3.5
Obstetrics and Gynecology	8
Pathological Anatomy	3.5
Pediatrics I	8
Psychiatry I	5
Surgery II	8
Urology	3.5
Year VI	
Community Medicine	6
Medicine III	16
Obstetrics	6
Pediatrics II	7
Psychiatry II	4
Surgery III	11
Research Project	7
Optional I, II	3
Total ECTS	180

## Abbreviations

AIC: Akaike information criterion
BIC: Bayesian information criteria
CI: Confidence interval
ECTS: European Credit Transfer and Accumulation System
ICC: Intraclass correlation coefficient
Log L: Log likelihood
PCV: Proportional change in variance
SH: % of study hours allocated in our curriculum to self-study hours
